# Psychomotor Impairment Detection via Finger Interactions with a Computer Keyboard During Natural Typing

**DOI:** 10.1038/srep09678

**Published:** 2015-04-16

**Authors:** L. Giancardo, A. Sánchez-Ferro, I. Butterworth, C. S. Mendoza, J. M. Hooker

**Affiliations:** 1 Madrid-MIT M+Visión Consortium, Research Laboratory of Electronics, Massachusetts Institute of Technology, Cambridge, MA 02139; 2 Athinoula A. Martinos Center for Biomedical Imaging, Department of Radiology, Massachusetts General Hospital, Harvard Medical School, Charlestown, MA 02129

## Abstract

Modern digital devices and appliances are capable of monitoring the timing of button presses, or finger interactions in general, with a sub-millisecond accuracy. However, the massive amount of high resolution temporal information that these devices could collect is currently being discarded. Multiple studies have shown that the act of pressing a button triggers well defined brain areas which are known to be affected by motor-compromised conditions. In this study, we demonstrate that the daily interaction with a computer keyboard can be employed as means to observe and potentially quantify psychomotor impairment. We induced a psychomotor impairment via a sleep inertia paradigm in 14 healthy subjects, which is detected by our classifier with an Area Under the ROC Curve (AUC) of 0.93/0.91. The detection relies on novel features derived from key-hold times acquired on standard computer keyboards during an uncontrolled typing task. These features correlate with the progression to psychomotor impairment (p < 0.001) regardless of the content and language of the text typed, and perform consistently with different keyboards. The ability to acquire longitudinal measurements of subtle motor changes from a digital device without altering its functionality may allow for early screening and follow-up of motor-compromised neurodegenerative conditions, psychological disorders or intoxication at a negligible cost in the general population.

In 1844 the first finger operated electrical device for long distance communication was invented. It was soon discovered that operators using this machine, the telegraph, were unwillingly disclosing more information than the message itself. Experienced telegraph operators were able to identify their colleagues by listening to patterns in the rhythm of Morse code being communicated^1^. During World War II, American intelligence developed a methodology called “The Fist of Sender” to distinguish telegraph messages coming from allies or enemies^2^. With the advent of the computer, the same identification concept was adapted to a computer keyboard. In 1983, J. Garcia filed the first patent describing a method able to identify a person via their style of typing on a computer keyboard^3^. In the last 30 years, many have proposed the development of pattern recognition algorithms to certify the identity of a person from typing-derived features^4^,^5^, now known as keystroke dynamics. Fig. 1 shows examples of time-based features commonly employed for user identification. Two recent reviews^4^,^5^ compare various keystroke dynamics classification methods, some of them achieve an excellent accuracy, with an identification rate higher than 95%. The main challenge that these methods face is the need of taking into account the user variability due to physical or psychological variations, an aspect reported in various publications^5^,^6^. Our hypothesis is that while physical or psychological variations may undermine accuracy for biometric applications, they may be leveraged to infer the psychomotor status of the subject typing.

Current algorithms are highly specialized for biometric identification and are not tuned to characterize health-related variations. The algorithms span a vast range of families: basic statistical features^7^,^8^,^9^, Bayesian analysis^10^,^11^, autoregressive models^12^, hidden Markov models^13^,^14^, artificial neural networks^15^,^16^ and other machine learning techniques^17^,^18^. Each of these approaches is employed to grant or deny access to a computer system, hence, a primary requirement is the reliance on a small number of key presses in order to avoid excessive burden on the user who needs to access the system. In light of these constraints, algorithms are typically tailored for recognizing typing patterns in known pre-defined text, which helps achieve a high classification performance. On the other hand, we want to extract relevant psychomotor-related information regardless of the text typed and without changing an individual's daily workflow. By employing software that operates in the background of one's daily activities, we would be able to monitor typing patterns longitudinally that can be used to infer or detect changes in health or state, especially considering the number of daily interactions one typically has with keyboards, touchscreen devices or other appliances.

Inverse kinematic analysis of the forces involved in key presses by means of high speed cameras in conjunction with force sensors^19^, electromyography^20^ and computer models^21^ identifies three phases for a single keystroke: key mechanism compression, finger impact, and fingertip pulp compression, followed by release. The key mechanism compression starts when the finger first reaches the contact with the key and ends when the key has been fully pressed, it accounts for ~12% of the total Hold Time (HT). When a key reaches full compression maximum finger deceleration and peak force occurs, this phase accounts for another ~11% of the hold time. Then, the tip of the finger moves down less than a millimeter due to the skin compression and it is finally released from the depressed key, this phase lasts for the remaining ~77% of the hold time. Interestingly, the duration of this phase is not correlated to the forces employed in the first two^19^; Jindrich et al.^22^ compared the finger tapping kinematics on four structurally different keyboards with three different hand postures, finding that kinematics, endpoint forces, net joint torques, and energy production showed similar patterns.

Previous studies aimed at explaining the neurobiology of typing and keystroke dynamics have revealed that hold times are generally very short, typically around 100 milliseconds^19^,^20^; still, keystrokes trigger both cortical and subcortical brain networks which have been identified by neuroimaging functional studies. Witt et al.^23^ compared and contrasted 38 independent studies (22 fMRI and 16 PET) solely on finger tapping in order to identify the brain activation areas. In all studies, the consistent areas activated were: primary sensorimotor cortex (SM1), supplementary motor area (SMA), basal ganglia (BG), and cerebellum. Additionally, clusters of activation were observed in the premotor and parietal cortices, these regions are known to play an important role in the transformation of sensory input to motor tasks, and the production of complex motor tasks. Thus, impairment related to these areas may be detectable via changes in typing patterns.

In order to test this hypothesis, we selected a condition known as “sleep inertia” as a proof of principle. This psychomotor effect has been described as a state of grogginess, impaired cognition, reduced motor dexterity, and disorientation while awakening^24^. Its impact on a subject performance is comparable to being sleep deprived for 24 hours^25^ or inebriated^26^,^27^. Although always present during awakening, the effect is maximal after the slow wave or deep sleep phases, lasting typically 15 to 30 minutes in healthy subjects^28^,^29^.

In this study we present a novel algorithm based on the evolution of key press latencies or hold time that is able to detect the psychomotor decline induced by sleep inertia in healthy subjects. Fig. 1 shows a graphical representation of hold time and other keystroke dynamic variables.

## Results

### Features Statistical Significance

We evaluated the statistical significance of the direction vector **v^Δ^** representing changes in different psychomotor states. The significance of the directionality is performed by a Rayleigh test for circular uniformity^30^. Such analysis is very specific for directional data and successfully employed in many scientific fields, ranging from physics to neuroscience^31^. Fig. 2 shows the circular histograms for the state changes and the p-values under the null hypothesis that the samples are uniformly distributed around a circle.

The direction of the changes between rested and sleep inertia were significant (p < 0.001) in both repetitions. All subjects showed a change falling in the same upper-right quadrant. The direction of the change among similar states was not statistically significant (p = 0.26 and p = 0.48).

### Classification

Fig. 3 shows the Receiver Operating Characteristic (ROC) curves resulting from our classifier in two datasets. We obtained areas under the ROC curve (AUC) of 0.93 and 0.91.

For comparison, we computed the ROC curves of the best classification achievable via “raw” variables: median key hold time and average key presses (i.e. typing speed). This theoretical classification upper bound was calculated by sweeping a decision threshold (i.e decision stump) through the dataset without splitting between training and testing sets. The AUC obtained were 0.76/0.79 for the median hold time in the two datasets; and 0.73/0.79 for the average typing speed in the two datasets.

The whole classification process, including feature computation, can be performed in real time via an unoptimized Python implementation on a Intel Core i5 3.3 GHz machine with 8 GB of memory.

## Discussion

In this study, we were able to create a new set of numerical features to detect a psychomotor change in 14 subjects due to the sleep inertia effect. These features were employed to train and test a machine learning classifier which achieved an AUC of 0.93/0.91, showing that the test performs consistently in two repetitions spread more than 7 days apart. These results suggest that a system to detect psychomotor impairment is feasible, sufficiently accurate and reliable via routine typing in a relatively unconstrained setting. The performance of our method outperformed the best classification achievable with basic statistics on hold time or average typing speed, thus showing the benefit of the additional analysis steps introduced with our method.

**Figure 1:**
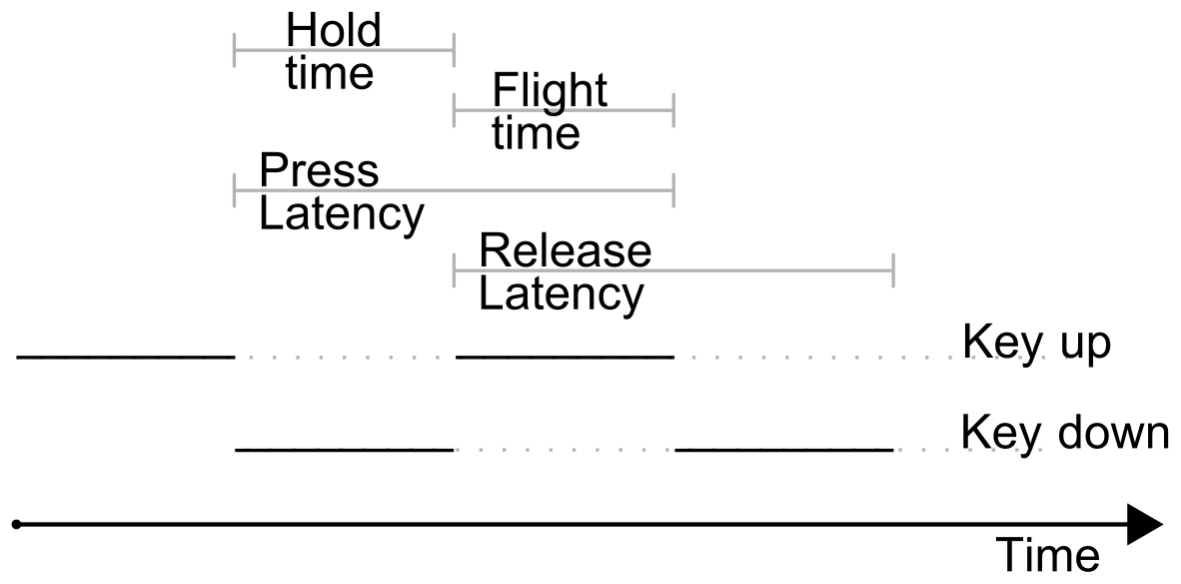
A pictorial representation of keystroke dynamics variables.

**Figure 2:**
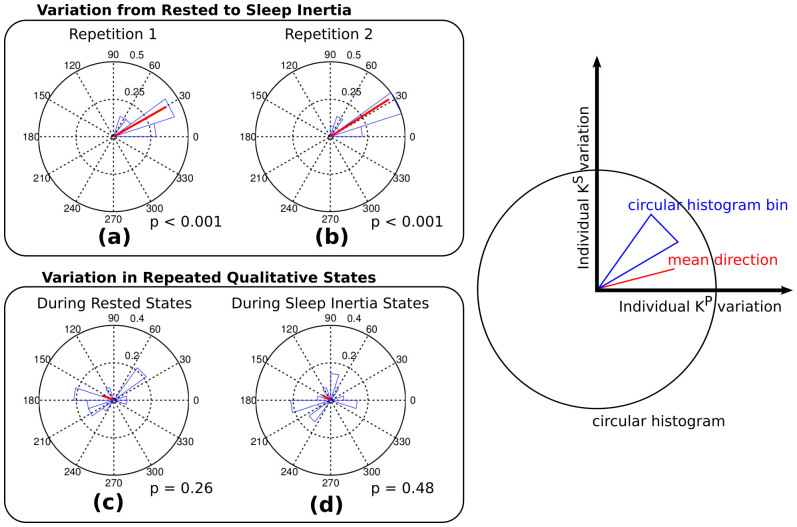
Circular histograms showing the directionality of v^Δ^ in variations of psychomotor impairment states. The p values of the Rayleigh test for circular uniformity under the null hypothesis that the samples are uniformly distributed around a circle assuming a von Mises distribution. Variations between rested and sleep inertia state (a,b) reject the null hypothesis, indicating a statistical significant direction in both repetitions. Variations in similar states (c,d) do not reject the null hypothesis indicating an insignificant directionality of the vectors.

**Figure 3:**
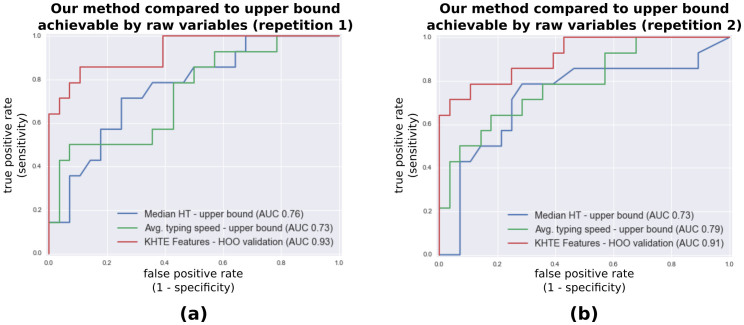
ROC analyses of the classification of the variation between rested and sleep inertia state vs. the variation during similar states. Our approach employing Key Hold Time Evolution (KHTE) features outperforms the upper bound classification achievable with median hold time (HT) and average typing speed in both repetitions. In the legend, the Area Under the ROC Curve (AUC) scores are shown. The KHTE features are classified by a linear Support Vector Machine tested via a hold-one-out (HOO) approach.

Acquiring a meaningful longitudinal signal from uncontrolled typing is a challenging task. The initial physiological parameters of a subject are not easily quantifiable, the text typed is completely unknown, and no assumptions about the typing style or number of pauses can be made. Despite the multitude of confounding factors in a subject's typing, it is reasonable to assume that on a modern machine, users will press and release a key as fast as they notice the end of the key travel (if mechanic) or as they perceive the tactile feedback of a touchscreen. In this study we concentrated our efforts on key hold time analysis, given that a number of factors indicated it as the most promising variable derived from the keystroke dynamics (shown in Fig. 1), that could be indicative of impaired brain functions. The reasons are twofold: firstly, the kinematics and forces involved in a finger pressing a key have been already studied in depth^19^,^20^,^21^,^22^ suggesting which are the underlying brain functions involved; secondly, the action of pressing a button is significantly less likely to be affected by text typed, typing speed or typing skills in comparison to flight time, release latency, press latency or number of keys typed.

Sleep inertia was the model of choice for several reasons. First, sleep inertia is straightforward to induce in healthy subjects. Its effects are time-limited and well described^32^ allowing for longitudinal evaluation of a participant under scrutiny. Second, sleep inertia influences motor tasks. This is based on the fact that the awakening process drives a sequential activation of different brain areas. Sensory-motor regions, such as SM1 and SMA, have a delayed activation when compared with anterior frontal and brainstem areas, as shown in functional imaging studies^33^. Additionally, the choice of sleep inertia for psychomotor impairment allowed us to capture data in the same real world setting where future tools implementing this technology are likely to be used.

An inherent limitation of the study design is the qualitative nature of the “rested state”: each subject was asked to type while they felt well rested, but we did not monitor exactly what was the subject's sleeping pattern in the preceding nights and numerically quantify the subject's fatigue. Similarly, the exact strength of the “sleep inertia state” is not known since we did not exactly monitor the sleep phase the subjects were in. In spite of these difficulties, the features derived from the key hold time evolution signal showed a clear directional trend from rested state to sleep inertia state, while no statistical significant directional trends were detected in repetitions of the same qualitative states. Sleep inertia is an effect that occurs in all phases of sleep, thus subjects had a nightly psychomotor impairment that is inevitably greater than the impairment of the rested state during the day. Our cohort included a diverse group of subjects in terms of mother tongue, writing language, keyboard used and computer model in order to maximize the heterogeneity of the population studied. Nonetheless, all of them were less than 40 years old and had prior exposure to technology. In further studies, we will evaluate the system in an elderly population with a lower computer literacy. Additionally, we will evaluate the influence of other variables such as: number of fingers employed, keyboard type and typing style.

Our results suggest that the finger interaction with electronic devices is capable of providing informative data about psychomotor status without changing the subject's daily workflow or the functionality of the device used. Quantify psychomotor function could provide an enormous quantity of longitudinal data that, to our knowledge, has never been explored for health care-related purposes. These data can be acquired entirely via a web browser making possible the mass deployment of what we refer as a “transparent” method, where the daily workow of a subject is used to infer health-related information.

From the perspective of the practical use of such a technology, our findings could enable the creation of safety software applicable to all activities that require taking important or potentially dangerous decisions during night shifts or other factors conditioning drowsiness^34^. Additionally, this new line of research has the broader potential of being applicable to other medical domains where a psychomotor change is expected; i.e. detection and progression monitoring of neurodegenerative diseases (such as Parkinson's and Alzheimer's disease), psychological disorders or intoxication with any substance affecting the central nervous system.

## Experimental Procedures

Fourteen subjects (7 males, 7 females) aged between 20 and 39 years (mean 30.8, standard deviation 4.4) participated in the study. None of the subjects was a professional typist but all self reported spending at least one hour per day using a computer. The typing style varied across subjects, however each subject self-reported consistency in style for each test. Table 1 shows the typing speed of each subject which is typically used as a measurement of typing abilities. The subjects were prescreened to exclude the following conditions: active cancer, neurodegenerative disorders (Parkinson's disease and Alzheimer's disease), hand articular problems (arthritis or osteoarthritis), severe liver or renal failure, active epilepsy (defined as one seizure within the last year). The other exclusion criteria were the daily consumption of any drug with sedative effect (antidepressants, hypnotics, antiepileptic, opioid analgesics, histamine antagonists). Alcohol consumption was not permitted on the day of the study. The subjects did not exactly know what was going to be measured, they were generally informed that we would be monitoring their typing patterns. Subjects gave informed consent prior to experiments, and experimental procedures were approved by COUHES (Committee On the Use of Humans as Experimental Subjects) at the Massachusetts Institute of Technology, protocol no. 1311.

We induced a subtle psychomotor impairment by waking each subject up during the night, thus inducing a sleep inertia status. The subject sent to the experimenters a text message just before going to sleep, then, they were woken up by a phone call 70–80 minutes after the time the message was received. This allowed ~20 minutes to fall asleep (sleep onset latency), and an additional 50–60 minutes which typically corresponds to the phase III/IV of a sleep cycle^35^. The test was considered invalid if subjects reported that were not able to fall asleep within 20 minutes (a single invalid trial was identified; the test was dismissed and the day/night repetition was retaken). Once awakened, subjects were asked to select a Wikipedia entry of their choice and type it on a web page for 15 minutes. The subjects were free to select any type of text as long as they had not typed it previously. Our intention was to limit any type of learning effect confounding our results. Also, they could type any type of language or machine they wanted, as long as they were consistent across all repetitions, see Table 1. In the table, the keyboard configuration, language typed, mother tongue and laptop size are also reported.

The subjects were tested once during the day, whenever the subject felt well rested, and then they were woken up during the night that immediately followed. The same pair of tests was repeated after at least a week at the subject discretion, see Table 1. By the end of our study, we were able to collect ~14 hours of key timestamps. All the typing was performed on the subject's personal computer.

### Data Acquisition

The keystroke dynamics were captured in the background and securely sent to a central server by a browser plugin developed by our research group. The plug-in ran entirely on the local machine and saved all the key timestamps to memory before submission to a central server, this to avoid any bottleneck due to network stability. The browser plugin was specifically designed for the latest version of Google Chrome available at the time of the experiments (March 2014). This allowed to make use of the internal high resolution timers and multi-threaded non-blocking calls to acquire key timestamps without relying on a fixed sampling rate. We have evaluated the timing resolution of our plugin by injecting a series of software generated key presses and releases into the operating system event queue. A stream of two consecutive events (key-down, key-up) was generated every 200 milliseconds for a total running time of 15 minutes. On Windows 8.1, we obtained a temporal resolution of 3.18/0.84 (mean/std) milliseconds with a 2.8 GHz Intel Core i5; on Linux (kernel 3.18), we obtained a temporal resolution of 3.25/0.46 (mean/std) milliseconds with a 2.8 GHz Intel Core i5; on MacOS (Mavericks), we obtained a temporal resolution of 0.82/0.51 (mean/std) milliseconds with a 2.6 GHz Intel Core i7.

Another aspect to take into consideration is that, if keystrokes are made fast in succession, they might not immediately appear on the screen because of the internal visual buffering of the web browser. We estimated that our setup running on Google Chrome had a visual buffering of ~100 milliseconds. Considering the typing speed of our subjects (Table 1), the visual buffering delay is unlikely to lead to keys stricken twice unintentionally.

During the signing of the informed consent, the subjects were instructed on how to temporarily disable all non-essential software, including operating system updates and resource intensive anti-viruses. This allowed to run the experiments avoiding processes that may delay feedback on the screen or lead to delayed timestamps measurements. In addition, the subjects typed on a bare form that did not require any intense computation, network or disk input/output during the typing task.

## Methods

We present a new type of signal representation based on the hold time time series, that we call Key Hold Time Evolution Matrix (K). Based on this we derive two numerical features that summarize with two numbers the large quantity of data captured in K. These features are then used to build a Machine Learning-based test that, given a reference in time when the subject is rested, can classify the psychomotor impairment caused by sleep inertia.

### Key Hold Time Evolution Matrix (*K*)

**Figure 4:**
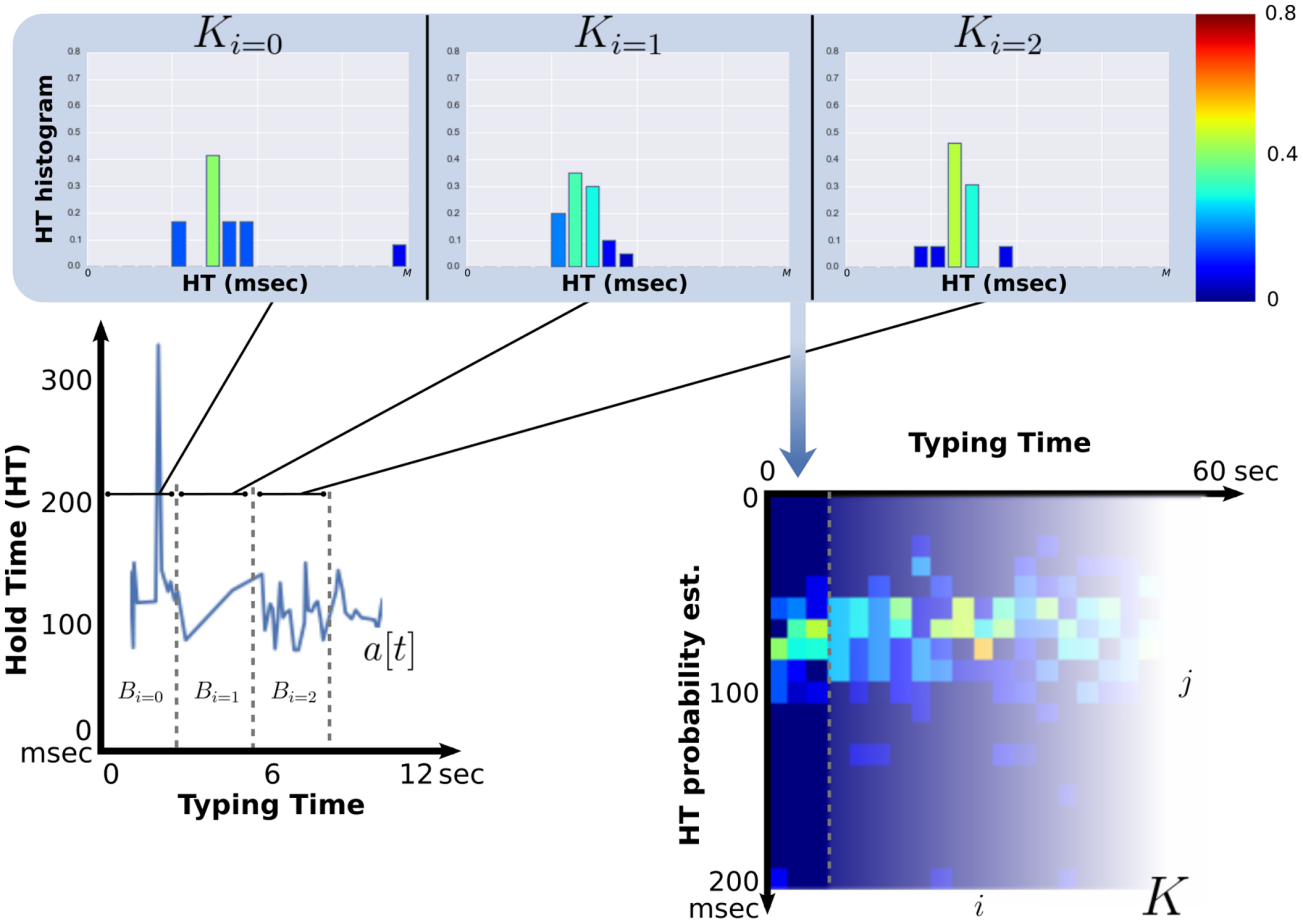
Process to derive the *K* matrix from the time series of key Hold Time (HT). The hold time signal *a*[*t*] is first divided by a square time window into vectors containing a variable number of hold time samples. These vectors are stored in the set *B* only if contain more than *S* samples. For each vector in *B* a normalized histogram is computed as an approximation of the hold time probability. Finally, the histograms are vectorized and used as columns of the matrix *K*.

**Figure 5:**
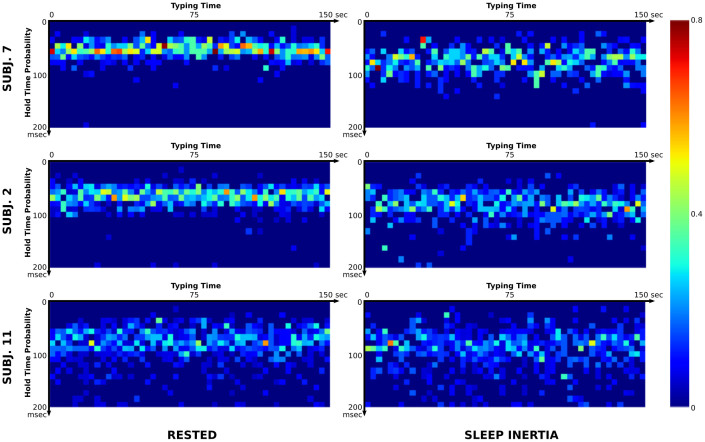
Examples of Key Hold Time Evolution matrices in rested subjects compared with a state of sleep inertia. The examples are chosen so that it can be appreciated how the change between the two states is at times visually obvious (top row), somehow visible (middle row) and hard to visualize (bottom row). The difference in the states was correctly detected by our algorithm in all three instances shown.

Let the vector *a*[*t*] represent continuous-time stochastic process of hold times where *t* is the time at which a key has been pressed to generate the relative hold time variable. We define a rectangular time window *ω*, such that:
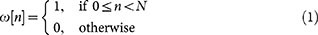
where N is the size of the window expressed in milliseconds. Then, it is possible to partition *a* into the time domain into a set *B* of vectors with varying length:
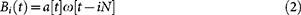
where *i* is a positive integral number which serves as an index to the list of vectors. In order to deal with the potential sparsity of raw hold time data, all the vectors in *B* that have a large number of zero elements are removed from the set. The minimum number of non-zero elements is defined by *Q*. Finally, let us define the matrix *K* as:
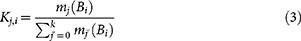
where *m_j_* is the number of observations that fall in the bin *j*. The bins are *k* equally spaced bouts from 0 to *M* milliseconds. The last bin also includes all the observation greater than *M*. Each column of the *K* matrix represents the normalized histogram of the hold time signal at the time window with index *i*, i.e. an estimation of the Probability Density Function (PDF). Fig. 4 graphically represents the process. Fig. 5 shows representative examples of Key Hold Time Evolution matrices in rested subjects compared with a state of sleep inertia.

### Compact Representation of *K*

The signal in *K* can be relatively large, even after a few minutes of typing. While this is an advantage in terms of the ability to capture the nuances in the hold time, it poses a problem for the quantitative interpretation and subject comparisons for both human experts and computer algorithms. In here, we introduce two new features, in the machine learning sense, that are able to provide a compact two-dimensional representation of *K*: Key Hold Time Evolution Peak (*K*^p^) and Key Hold Time Evolution Self Similarity (*K*^s^). The Key Hold Time Evolution Peak (*K*^p^) captures the main mode of each column in *K* and averages them as follows:
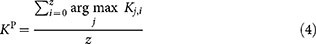
where *z* is the number of columns of the *K* matrix. The intuition behind this measurement is that subjects with sleep inertia tend to show an increased hold time, as shown in the first row of Fig. 5. Since no assumption about the distribution can be safely made, this approach allows a robust estimation even when only few data points are available in the time window. Another approach would be to use a large value for *Q*, hence discarding all *B* that do not have enough information, which allows the estimation of a real valued PDF. In our experience, this would have required us to force subjects to type at a substantial speed to capture enough data, which would limit our ability to create a transparent tool that monitors the uncontrolled typing. It was observed that the stability of each column in *K* across time provided a strong indication of sleep inertia status. Higher instability was found for the sleepy subjects, as shown in Fig. 5. We wanted to capture this information into features that can be independent of typing pauses and distribution of the PDFs. Thus, we computed a self-similarity matrix *S* that characterizes *K* across time. Among the different distance metrics proposed in the literature, given our choice of the *L*_2_ norm (Euclidean distance), we can compute the time self similarity matrix *S* as follows:
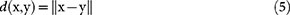

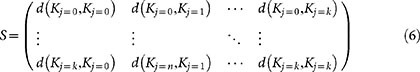


**Figure 6:**
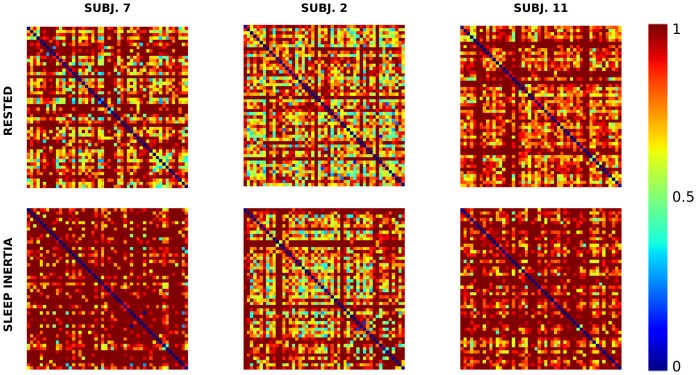
Examples of *K*^s^ derived from the signals shown in Fig.5. The more the matrix tends to red, the less each time window is similar to others (i.e. less connected).

Then the matrix *S^i^* is obtained by normalizing *S* between 0 and 1 where 1 corresponds to the scalar parameter *L*. Fig. 6 shows some examples of such self similarity matrices. These matrices can be further reduced to a multitude of measures by leveraging standard network theory. We are currently employing a measure of global connectivity that we call Key Hold Time Evolution Self Similarity (*K*^s^):
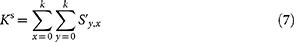


### Feature Vector and Classification

In order to detect a state of impairment or lack thereof, we create a reference feature vector **v** = (*K^P^*, *K^S^*)*^t^* and a second vector 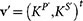. Their difference is another 2-dimensional vector **v^Δ^** that indicates the change of psychomotor status of the subject at the two time points. All the parameters to calculate *K^P^* and *K^S^* have been estimated by maximizing the norm of **v^Δ^** where **v** is rested state at repetition 1 and **v**′ is sleep inertia state at repetition 1 by gradient descent. The optimizer did not have any information about the direction of the change and the experimental data at repetition 2. Given the novelty of the signal and its features, we performed an ANOVA-like circular statistical analysis on the significance of the direction of the change represented by **v^Δ^**, see Results section.

The decision boundary of psychomotor impairment signs was learned by example via a Support Vector Machine with a linear kernel and probability estimation as implemented in libSVM^36^. The classifier is trained and tested via a hold-one-out approach on two different datasets. The first dataset contains: the positive **v^Δ^** samples encoding the difference between rested and sleep inertia state in each day/night pair (i.e. a significant change in the psychomotor status of the subject) in the first repetition. Negative **v^Δ^** samples encoded the difference among the same states across the two repetitions (i.e. changes in the psychomotor status of the subject whose magnitude is less than the positive samples). The second dataset contained the same negative samples and positive samples measured in the second repetition.

## Supplementary Material

Supplementary Information Supplementary info

## Figures and Tables

**Table 1:** Subject Characteristics

Subject	Gender	Age	Days between Repetitions	Operating System	Laptop Size	Keyboard Type	Language Typed	Mother Tongue	Avg. Typing Speed
1	Male	29	7	MacOS	15	Swiss French	English	French	243 (12.3)
2	Male	30	34	Windows	14	US English	Spanish	Spanish	248 (44.7)
3	Female	29	15	Windows	14	US English	English	Bulgarian	263 (14.7)
4	Female	20	8	Windows	14	US English	English	English	177 (28.6)
5	Male	32	9	MacOS	N/A	US English	N/A	Spanish	281 (15.3)
6	Male	28	24	MacOS	13	US English	English	English	269 (46.1)
7	Male	32	9	Windows	13	Spanish	Spanish	Spanish	195 (26.3)
8	Female	30	15	MacOS	13	Spanish	Spanish	Spanish	180 (23.0)
9	Female	30	42	Windows	11	Turkish	Turkish	Turkish	164 (19.6)
10	Female	38	8	Windows	15	Spanish	English	Spanish	126 (13.2)
11	Male	39	8	Windows	12	US English	English	Spanish	234 (27.7)
12	Female	32	12	MacOS	13	US English	Spanish	Spanish	261 (38.7)
13	Male	33	20	MacOS	13	Spanish	Spanish	Spanish	132 (30.1)
14	Female	30	21	Windows	13	US English	English	Spanish	240 (20.9)

(N/A) Not Available.

Typing speed is measured in average characters per minute. The standard deviation is shown between the parentheses.
