# Supplementary material for: Psychomotor Impairment Detection via Finger Interactions with a Computer Keyboard During Natural Typing
Source: Sci Rep. 2015 Apr 16;5:9678. doi: 10.1038/srep09678 (PMC5381750; doi:10.1038/srep09678)
Supplement: Supplementary Information — Supplementary info [file srep09678-s1.pdf]

Psychomotor Impairment Detection via Finger Interactions with a Computer Keyboard During Natural Typing

L. Giancardo, A. Sanchez-Ferro, I. Butterworth, C. S. Mendoza, J. M. Hooker

Additional material

The datasets are available at the following address:
https://www.neuroqwerty.com/paperSI/datasets.zip

The form used for the experiment is viewable here:
https://www.neuroqwerty.com/paperSI/sleep_test/

The plugin and source code to acquire the data is downloadable here:
https://www.neuroqwerty.com/paperSI/sleep_test/chromeExt-v0_3.crx
